# Potential adaptive habitats for the narrowly distributed and rare bamboo species *Chimonobambusa tumidissinoda* J. R. Xue & T. P. Yi ex Ohrnb. under future climate change in China

**DOI:** 10.1002/ece3.70314

**Published:** 2024-09-15

**Authors:** Wei‐Hua Wang, Shu‐Lei Peng, Hua Shu, Xi Fu, Xia‐Ying Ye

**Affiliations:** ^1^ Agronomy and Life Science Department Zhaotong University Zhaotong Yunnan China; ^2^ College of Agronomy and Biotechnology Yunnan Agricultural University Kunming China

**Keywords:** bamboo, *Chimonobambusa tumidissinoda*, climate change, environmental variables, MaxEnt model, suitable habitats

## Abstract

The global climate change has resulted in substantial modifications to the distribution patterns of narrowly distributed species across different time periods, leading to an imminent threat to the survival of some vulnerable species. *Chimonobambusa tumidissinoda* J. R. Xue & T. P. Yi ex Ohrnb., a bamboo species endemic to the transition zone from the Yunnan‐Guizhou Plateau to the Sichuan Basin with high economic and ecological value, exhibits a limited range and rarity. Utilizing eight environmental variables and 56 occurrence records, we employed the MaxEnt model to predict the potential distribution range of *C. tumidissinoda* under current and future climate scenarios. The findings revealed that precipitation of the driest month (Bio14), elevation and isothermality (Bio3) were the crucial factors determining the species' distribution, accounting for 31.24%, 28.27% and 17.24% of data variability, respectively. The distribution centroid of *C. tumidissinoda* is anticipated to shift towards higher latitudes in response to future climate change, and the projected habitat suitability is expected to expand under ssp245 and ssp585 scenarios while remaining unchanged or contracting under the ssp126 scenario. Despite these expansions, the suitable habitats remain limited, with the largest being approximately 2.08 × 10^4^ km^2^, indicating a significant threat to its survival. Our study provides insights into the adaptive responses of *C. tumidissinoda* to climate change, enriching scientific knowledge for developing effective conservation measurements as well as sustainable utilization.

## Graphical abstract

Chimonobambusa tumidissinoda is a rare and narrowly distributed bamboo species, we utilized MaxEnt model to predict its potential distribution range and evaluate its vulnerability under future climate scenarios. Simulation results revealed that although the suitable area for C. tumidissinoda is projected to expand under two climate scenarios, the habitat range of this bamboo remains limited across various scenarios with precipitation and elevation being the primary influencing factors. Therefore, more emphasis should be placed on its conservation efforts.

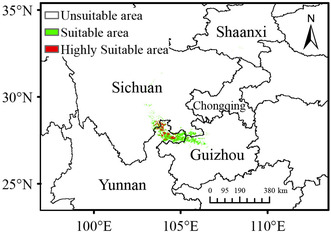

## 1 INTRODUCTION

The sixth assessment report of the Intergovernmental Panel on Climate Change (IPCC) highlights that the global temperature of 2011–2020 was approximately 1.1°C warmer than that of 1850–1900. And this warming trend is continuing, with projections indicating a temperature rise exceeding 1.5°C by 2100 in spite of the implementation of low‐emission technologies (IPCC, 2023). The dramatic climate change plays a crucial effect on shaping the distribution of plants and acts as one of the main drivers for habitat fragmentation and species extinction (Qiu, Fu, et al., 2023; Rong et al., 2023; Wang et al., 2024), especially for narrowly distributed and/or endangered species (Ohlemuller et al., 2008; Tang et al., 2022; Zhang et al., 2024). These impacts are becoming increasingly significant on a global scale, resulting in the loss of biodiversity and degradation of ecosystems. Therefore, it is essential to understand how these species will respond to future climate change and the probability of extinction for developing effective conservation measurements and maintaining biodiversity.

*Chimonobambusa tumidissinoda* J. R. Xue & T. P. Yi ex Ohrnb., commonly known as Qiongzhu or Luohanzhu, belongs to the genus *Chimonobambusa* of the tribe Arundinarieae (Poaceae, Bambusoideae) (Li et al., 2006). This bamboo species is a perennial herbaceous plant primarily propagated clonally through rhizomes and exhibits an extended sexual reproductive cycle (approximately 40 years, Ye et al., 2024), which may render it more susceptible to the impacts of climate change. The distribution range of *C. tumidissinoda* is limited, confined solely to the transition zone from the Yunnan‐Guizhou Plateau to the Sichuan Basin. As a result of this restricted distribution, it has been classified as a rare and endangered species since 1991 (Fu, 1991). This species possesses significant ecological and economic value. It is characterized by its occurrence in cloud‐shrouded hilltops at elevations ranging from 1300 to 2200 m. Moreover, it acts as an essential component of wet evergreen broadleaved forest in China (Keng & Wang, 1996). This plant is commonly utilized for handicraft production and cultivated as an ornamental plant due to its distinctive disk‐shaped nodes and elegant morphology. Since the Han Dynasty, the culms of this graceful species have been utilized as walking sticks and presented to foreign guests as prestigious gifts. However, previous study has indicated that *C. tumidissinoda* has undergone severe population degradation due to excessive human interference (Dong, 2006), and the plant growth could be affected by different environmental conditions, such as temperature, soil water and nutrition (Dong et al., 2002; Wu et al., 2019; Wu et al., 2020). Moreover, population genomics has revealed that this bamboo species exhibits a limited level of genetic diversity, indicating its vulnerability to future climate change (Ye et al., 2024). The findings of these aforementioned studies suggest that climate change has the potential to exacerbate its endangerment status, necessitating efforts to elucidate its future distribution trend. Currently, research on *C. tumidissinoda* mostly focus on its bamboo shoots (Wang et al., 2023; Yang et al., 2015; Yuan et al., 2008), genetic structure and diversity (Dong et al., 2016; Qiu et al., 2017; Ru et al., 2010; Ye et al., 2024), gene cloning and expression (Dai et al., 2023; Gao et al., 2021; Li et al., 2021), and physiological and community characteristics (Jia & Wang, 2021; Wang et al., 2012; Zhang et al., 2020; Zheng et al., 2021; Zhong et al., 2023). The research on the geographic distribution of this species under future environmental conditions remains insufficient.

With the rapid development of spatial distribution technology, species distribution models (SDM) are widely used to predict potential geographic ranges of species under current or future environmental scenarios based on their geographic distribution information (Guisan & Zimmermann, 2000; Vasconcelos et al., 2011). The prediction can provide valuable insights for assessing the ecological crisis and serve as a fundamental basis for biodiversity conservation. Among various SDM methods, the maximum entropy (MaxEnt) modeling stands out due to its exceptional performance and widespread utilization (Elith et al., 2006; Phillips et al., 2006). It employs a machine‐learning approach with presence‐only records to evaluate the potential distribution range of species. Furthermore, it exhibits superior accuracy compared to other SDM tools and have a better performance even when confronted with limited geographic distribution information (Elith et al., 2006; Phillips et al., 2006; Shi et al., 2023). In recent decades, the MaxEnt model has been extensively applied in the prevention of the invasion of alien species (Blackburn et al., 2020; McCulloch‐Jones et al., 2023; Tu et al., 2021), the protection of rare and endangered species (Gao et al., 2022; Tang et al., 2022; Zhang et al., 2023), the management of diseases and pests (Rawien & Jairam‐Doerga, 2022; Zhou et al., 2021), the evaluation of protected areas (Li et al., 2023; Thapa et al., 2018), etc.

In this study, we employed the MaxEnt model to evaluate the potential suitable areas for *C. tumidissinoda* in China under current and six future climate scenarios, based on its geographic distribution records along with bioclimate, topography and soil factors. Our objectives encompass: (1) identifying the predominant environmental factors influencing the distribution of *C. tumidissinoda* in China; (2) predicting the suitable distribution range of *C. tumidissinoda* in China across different time periods and climate scenarios; (3) elucidating the temporal dynamics of the potential suitable areas for *C. tumidissinoda* from present to future. This research provides invaluable insights for scientific exploration and conservation efforts for this narrowly distributed and rare bamboo species, *C. tumidissinoda*.

## 2 MATERIALS AND METHODS

### 2.1 Species distribution data and processing

The geographic distribution records of *C. tumidissinoda* were mainly obtained from two sources: (1) our field survey between 2020 and 2023 covering its distribution range and (2) specimen records from the Chinese Virtual Herbarium database (CVH: https://www.cvh.ac.cn/), the Global Biodiversity Information Facility (GBIF; https://www.gbif.org), the National Specimen Information Infrastructure (NSII: https://www.nsii.org.cn/), and published literature (Dong et al., 2016). The records were filtered to exclude those with duplicate coordinates, obvious identification errors, and unreliable distribution beyond its designated range. For records lacking geo‐coordinates but having specific locations, Baidu map was utilized to estimate approximate coordinates based on the geographic information provided in the specimen.

**FIGURE 1:**
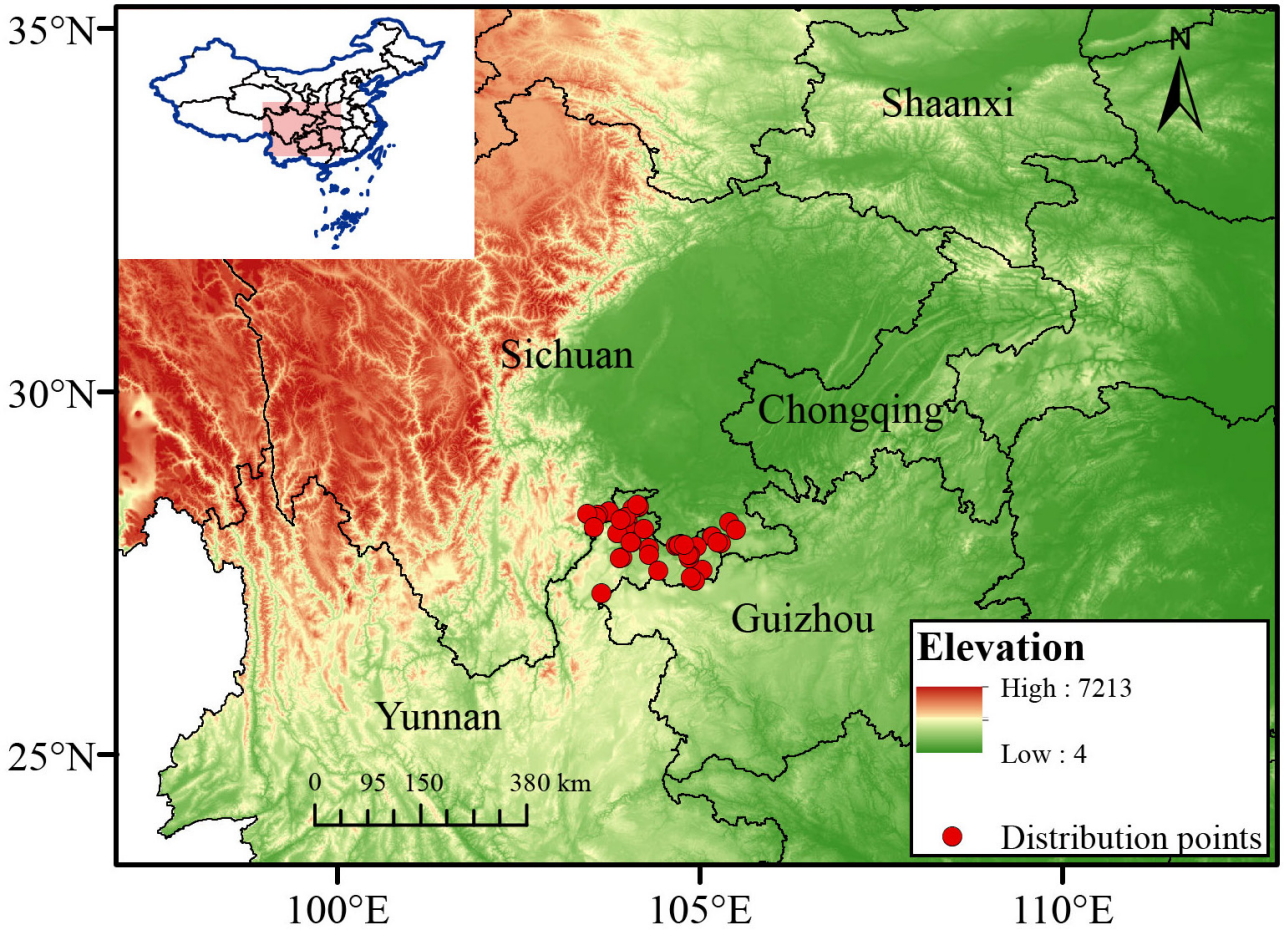
The study area and sampling records of *Chimonobambusa tumidissinoda*.

To reduce the impact of spatial autocorrelation, we employed SDM Toolbox v2.5 (Brown, 2014) in ArcGIS 10.7 to establish a buffer zone with a grid size 1 × 1 km (30 s), which is consistent with the resolution of bioclimatic variables utilized in this study. Subsequently, only one occurrence point was retained within each grid cell. Following filtering procedures, we obtained a final dataset consisting of 56 distribution records (Figure 1; Table S1). To meet the requirements of MaxEnt software, we stored the occurrence records in Excel and converted them into “.csv” format for future modeling.

### 2.2 Environmental variables and processing

We selected bioclimate, topography and soil factors to simulate the distribution pattern of *C. tumidissinoda* under current and future climate scenarios. We downloaded 19 bioclimate variables of near‐current (1970–2000) and two future periods (2041–2060/2050s and 2080–2100/2090s) from the WorldClim v2.1 (https://worldclim.org/) at a 30 arc‐second resolution (approximately 1 km^2^) (Fick & Hijmans, 2017). The future climate data were generated based on the BCC‐CSM2‐MR (Beijing Climate Center Climate System Model) climate system derived from the Coupled Model Intercomparison Project 6 (CMIP6). To provide a comprehensive comparison, three Shared Socioeconomic Pathways (SSPs) were selected for the future time periods: ssp126 (low forcing scenario with radiative forcing reaches 2.6 W/m^2^ in 2100), ssp245 (medium forcing scenario with 4.5 W/m^2^) and ssp585 (high forcing scenario with 8.5 W/m^2^). Subsequently, we acquired the elevation data from the Geospatial Information Authority of Japan (https://globalmaps.github.io/el.html). Slope and aspect were extracted from the elevation data in ArcGIS 10.7. Soil factors were obtained from the Harmonized World Soil Database v2.0 (https://www.fao.org/soils‐portal/). It assumed that both topographic and soil factors remained constant throughout the future period. All environmental variables layers were resampled uniformly at a resolution of 30 arc‐second and rasterized to match the same boundaries, cell size and coordinate systems as the occurrence data layer.

In order to address the issue of multicollinearity among variables and reduce model overfitting, we conducted a correlation analysis using Pearson's method on the 56 environmental factors. Firstly, bioclimatic factors with a contribution percentage greater than 1.0% (Qiu, Jacquemyn, et al., 2023) were identified through the pre‐simulation of all environmental factors in MaxEnt v3.4.4. Secondly, if the pairs of selected environmental variables exhibited an absolute Pearson's r value exceeding 0.8, the factor with a higher percent contribution was chosen. Finally, eight environmental factors were included in the further ecological niche modeling (Table 1).

**TABLE 1:** Environmental variables ultimately involved in MaxEnt modeling.

Category	Variable	Description	Unit
Climate	Bio3	Isothermality (BIO2/BIO7) (×100)	%
	Bio7	Temperature Annual Range (BIO5‐BIO6)	°C
	Bio14	Precipitation of Driest Month	mm
	Bio18	Precipitation of Warmest Quarter	mm
Topography	Elevation	Elevation	m
	Slope	Slope	°
Soil	Alum_sat	Aluminum saturation	%
	Gypsum	Gypsum content	%

### 2.3 MaxEnt modeling and evaluating

The potential spatial distributions of *C. tumidissinoda* were simulated using MaxEnt v3.4.4 based on 56 effective occurrence records and selected environmental variables. In this study, a random test percentage of 25% was used to verify the model's performance, while the remaining served as the training data. The regularization multiplier was set to 1, and the feature combination was automatically determined. We conducted 10 replicates of training to minimize the impact of random aberrations. Additionally, we utilized the jackknife method with options for “create response curves” and “Do jackknife” to assess the relative significance of each environmental factor. The final results were calculated from the average of 10 replicates and exported in ASCII format.

The accuracy of the simulated distribution models was evaluated based on the Area Under Receiver‐Operating Characteristic Curve (AUC) (Radosavljevic & Anderson, 2013). AUC values ranged from 0 to 1, with higher values indicating greater confidence in the prediction results. The category of AUC values are usually as follows: failed (AUC ≤0.6), poor (0.6 < AUC ≤ 0.7), fair (0.7 < AUC ≤ 0.8), good (0.8 < AUC ≤ 0.9), and excellent (0.9 < AUC ≤1) (Swets, 1988).

### 2.4 Classification of suitable areas

To determine the suitable and unsuitable regions of *C. tumidissinoda*, the resulting file was imported into ArcGIS 10.7 software and reclassified based on the 10‐percentile training presence logistic threshold value (TH) output by MaxEnt (Hughes, 2017; Radosavljevic & Anderson, 2013). According to the average TH value and the classification criteria outlined by IPCC (Mastrandrea et al., 2010), we categorized the potential distribution areas into three distinct groups based on their suitability levels (the Habitat Suitability Index, HSI): unsuitable area (HIS < TH), moderately suitable area (TH < HSI < 0.66), and highly suitable area (HIS ≥0.66), as previously employed in relevant studies (Qiu, Jacquemyn, et al., 2023; Shi et al., 2021; Yan, Gu, et al., 2021).

### 2.5 Species potential distribution change and centroid shifts

We utilized ArcGIS 10.7 to calculate the area of suitable habitat for *C. tumidissinoda* across different climate periods. Furthermore, the trend of changes in suitable habitat under various emission scenarios was assessed in comparison to the current condition. The contraction, expansion, and unchanged areas were determined using the “distribution changes between binary SDMs” tool from the SDM Toolbox v2.5 (Brown, 2014). Subsequently, we predicted the centroid location of the distribution area and identified the direction of shift between the current period and future climate scenarios (ssp126, ssp245 and ssp585 of 2050s and 2090s, respectively) using the “centroid changes clines” tool.

## 3 RESULTS

### 3.1 Model accuracy and potential current habitat distribution

**FIGURE 2:**
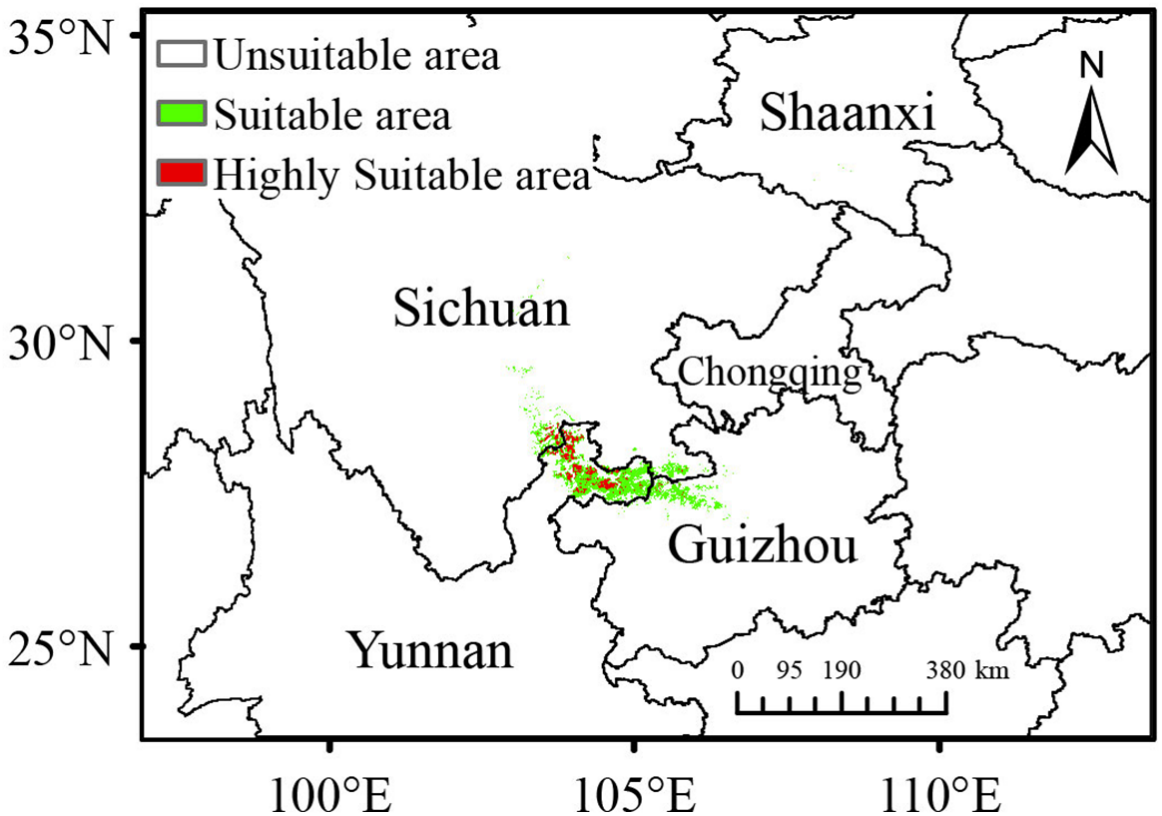
Potential distribution habitats for *Chimonobambusa tumidissinoda* under current climate scenario.

The average AUC values for all models of *C. tumidissinoda* ranged from 0.991 to 0.995 (Table 2), indicating exceptional model performance. The projected suitable area for *C. tumidissinoda* under the current climate scenario closely aligns with its existing physical distribution, with only 3.57% of occurrence records found in unsuitable area (Figure 2). The predominant concentration of these suitable habitats is primarily located in Zhaotong, Yunnan province and its adjacent area in Sichuan and Guizhou province, encompassing a relatively small range of 1.13 × 10^4^ km^2^ (Table 3). Within the suitable area, the proportion of highly suitable area only accounts for 18.58%, indicating a scarcity of areas that provide optimal conditions for *C. tumidissinoda*.

**TABLE 2:** AUC mean value of the simulated model of *Chimonobambusa tumidissinoda* under current and six future climate scenarios.

	Current	2050s			2090s		
		ssp126	ssp245	ssp585	ssp126	ssp245	ssp585
AUC	0.995	0.994	0.991	0.994	0.995	0.993	0.994

**TABLE 3:** Area (×10^4^ km^2^) and proportion (%) of suitable habitats for *Chimonobambusa tumidissima* under current and future climate scenarios.

Period	Moderately suitable are (%)	Highly suitable area (%)	Total suitable area (%)	Unsuitable area (%)
Current (1970–2000)	0.92 (0.49)	0.21 (0.11)	1.13 (0.60)	187.80 (99.40)
ssp126_2050s	0.98 (0.52)	0.16 (0.08)	1.14 (0.60)	187.79 (99.40)
ssp245_2050s	1.82 (0.96)	0.26 (0.14)	2.08 (1.10)	186.86 (98.90)
ssp585_2050s	1.03 (0.55)	0.26 (0.14)	1.29 (0.68)	187.65 (99.32)
ssp126_2090s	0.82 (0.43)	0.22 (0.12)	1.04 (0.55)	187.90 (99.45)
ssp245_2090s	1.10 (0.58)	0.21 (0.11)	1.31 (0.69)	187.62 (99.30)
ssp585_2090s	1.08 (0.57)	0.26 (0.14)	1.34 (0.71)	187.59 (99.30)

### 3.2 Key environmental factors affecting the distribution of *C. Tumidissinoda*

**FIGURE 3:**
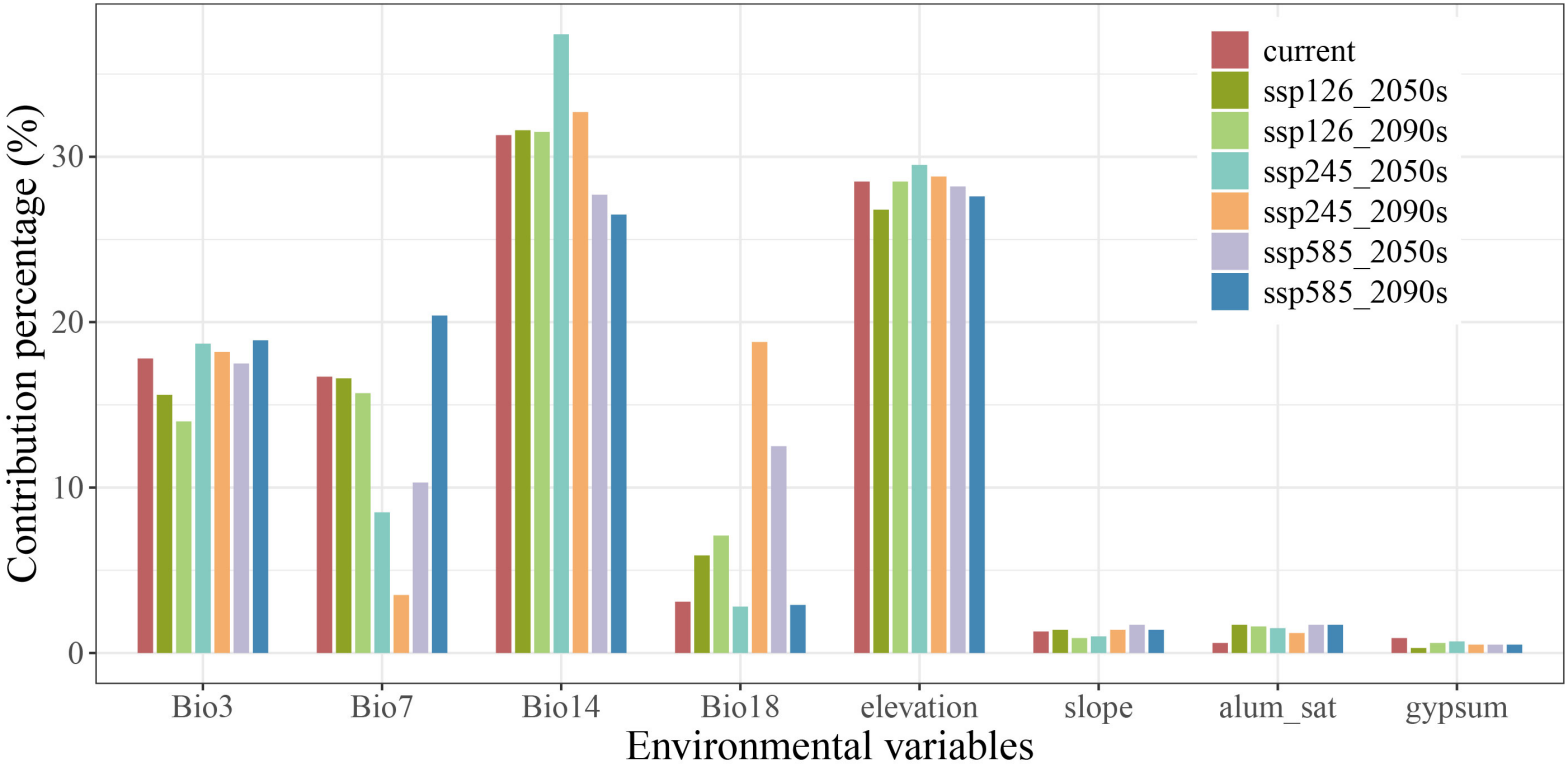
Contribution percentage of selected environmental variables on the distribution of *Chimonobambusa tumidissima*.

Based on the percentage contribution of environmental variables generated by the MaxEnt model (Figure 3), the geographic distribution of *C. tumidissinoda* is primarily determined by precipitation of driest month (Bio14, averaging 31.24%) and elevation (averaging 28.27%), followed by isothermality (Bio3, averaging 17.24%), which collectively account for approximately 77% of the predictive power of the model. The distribution of this bamboo species is also influenced by two other bioclimate factors (Bio7 and Bio18), which contribute approximately 20% in total. However, the proportion of these factors varies across different scenarios (Figure 3).

**FIGURE 4:**
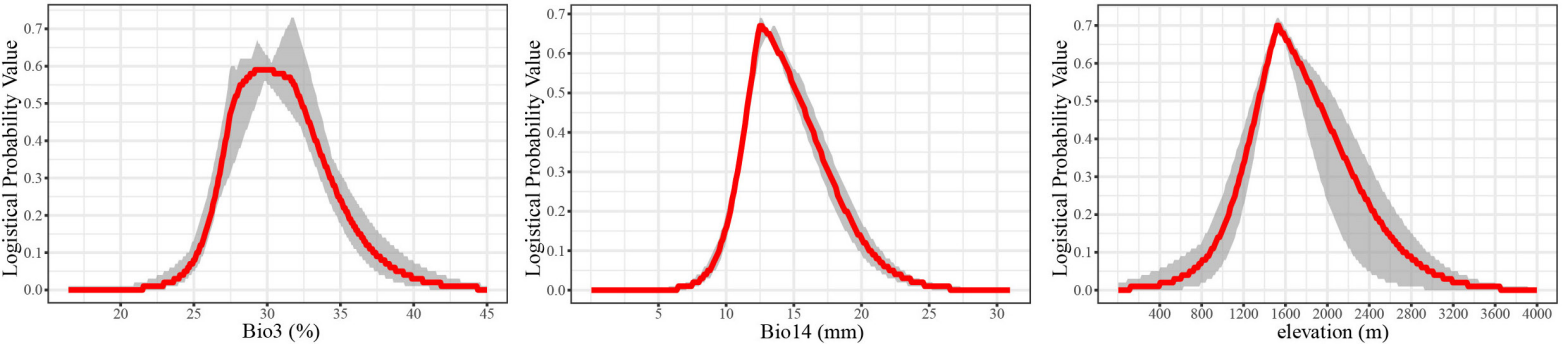
Response curves of key environmental variables based on individual variables. The red line represents the mean value, and the gray area indicates the range between the minimum and maximum values.

The response curves of key environmental factors can provide valuable insights into the relationship between the predicted occurrence probability and environmental variables, thereby elucidating the influence of each variable on the distribution pattern of *C. tumidissinoda*. The environmental factors are typically deemed conducive to plant growth when their probability of existence exceeds 0.5 (Fang et al., 2024; Yan, Wang, et al., 2021). The red lines depicted in Figure 4 illustrate the isolated impact of specific environmental factors on the projected probability of occurrence. Specifically, precise thresholds are necessary for the suitable distribution of *C. tumidissinoda*, including an elevation range of 1343−1907 m, an isothermality range of 27.69%–32.35%, and a precipitation range of 11.80–15.23 mm during the driest month (Figure 4).

### 3.3 Potential adaptive habitats of *C. Tumidissinoda* under future climate scenarios

**FIGURE 5:**
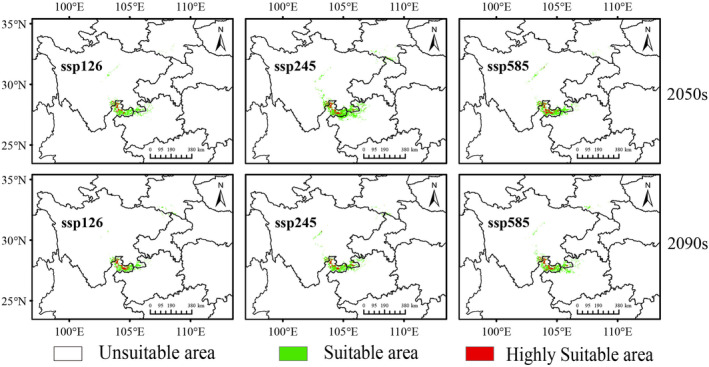
Potential suitable areas for *Chimonobambusa tumidissima* under future climate scenarios. The 2050s represents 2040–2060s period and the 2090s represents 2080–2100 s period.

The potential adaptive range of *C. tumidissinoda* under future climate scenarios (ssp126, ssp245 and ssp585) for the 2050s (2041–2060) and 2090s (2080–2100) was predicted in this study. The suitable habitat for *C. tumidissinoda* continues to primarily concentrate in Zhaotong, Yunnan province, and its adjacent regions with some variations compared to the current scenario (Figure 5).

Under the scenario of ssp126, the total suitable areas for *C. tumidissinoda* show limited changes, with only a 100 km^2^ expansion projected in the 2050s (Table 3). However, there is an observed degradation of highly suitable areas into moderately suitable ones at a rate of 23.81%, indicating a loss of preferred habitat of this bamboo species within this timeframe. In the 2090s, both the moderately suitable and total suitable areas experience reductions, with a slight decrease of 1.6 × 10^3^ km^2^ (16.33%) and 1.0 × 10^3^ km^2^ (8.77%), respectively, compared to the period in the 2050s.

Under the scenario of ssp245, there is a substantial growth of 9.5 × 10^3^ km^2^ (84.07%) in the total extent of suitable areas by 2050s compared to the current size, with nearly a doubling rise (197.83%) of moderately suitable areas and an expansion of highly suitable areas to 2.6 × 10^3^ km^2^ (123.81%) (Table 3). In contrast, during the 2090s, the total suitable area contracts to only 62.98% of its previous region in the 2050s, with both highly suitable areas and moderately suitable areas experiencing notable contractions. When compared to its current size, there is an expansion of approximately 19.57% in the overall extent of suitable areas.

Under the scenario of ssp585, the total habitat suitable for *C. tumidissinoda* shows a gradual expansion over time, reaching 114.16% of its current size by the 2050s and further increasing to 118.58% by the 2090s (Table 3). In terms of the highly suitable areas, they expand to cover an area of 2.6 × 10^3^ km^2^ (123.81%) in the 2050s and maintain stability thereafter. The moderately suitable areas exhibit a gradual increase over time as the total suitable areas, with their size reaching 111.96% by the 2050s and expanding further to 117.39% by the 2090s compared to their current size.

### 3.4 Range changes between current and future climate scenarios

**FIGURE 6:**
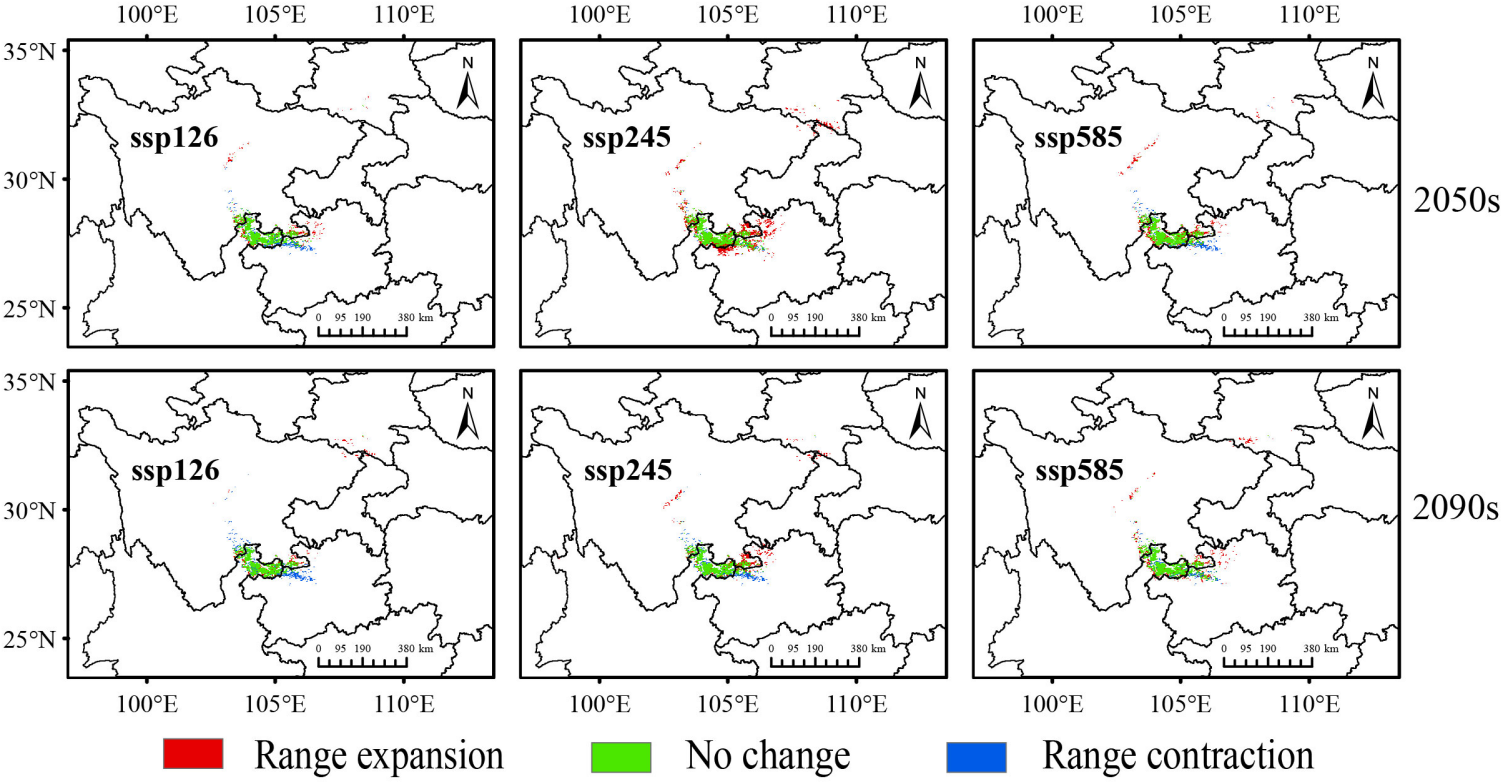
Potential habitat changes of *Chimonobambusa tumidissima* from current to future climate scenarios.

**FIGURE 7:**
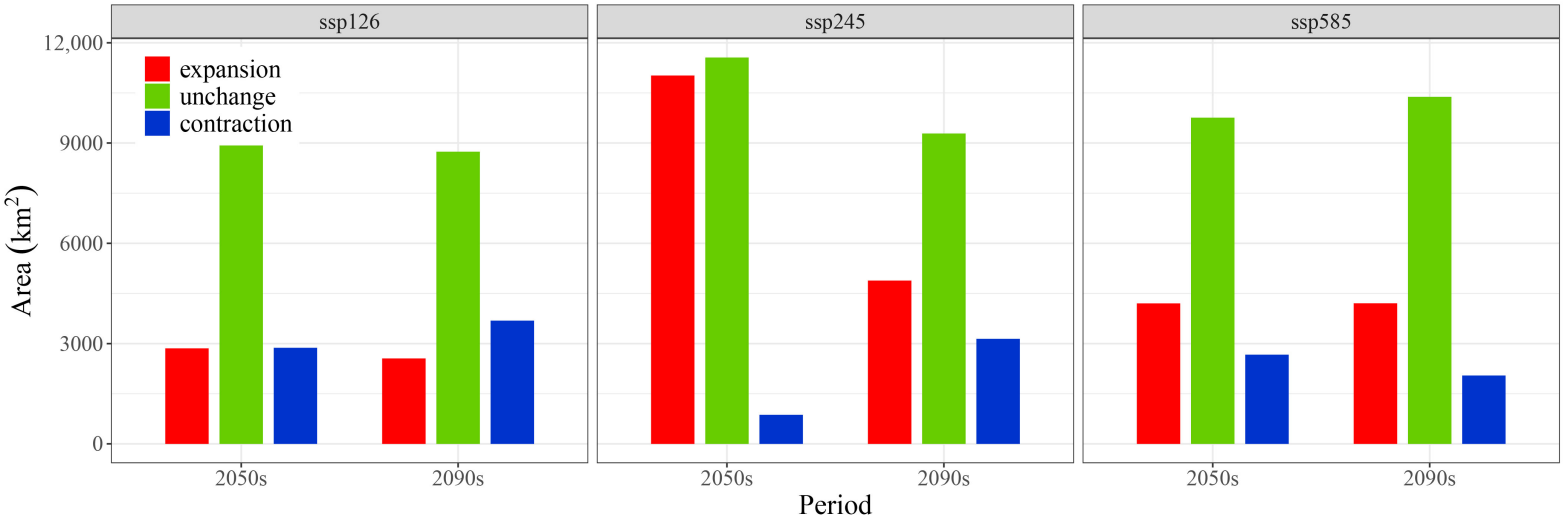
Changes of suitable habitat areas of *Chimonobambusa tumidissima* from current to future climate scenarios. Red represents potential range expansion, blue represents potential range contraction, and green represents the overlap of current and future projected ranges.

The potential distribution shifts of *C. tumidissinoda* are illustrated in Figure 6. The most significant contraction of suitable areas mainly occurs in Guizhou province under the ssp126 scenario of the 2090s (3686 km^2^, Figure 7). The stable area, predominantly encompassing Zhaotong in Yunnan province, continues to present the largest proportion of distribution areas and exhibits a significant stability under the ssp245 scenario in the 2050s (11,573 km^2^). The expansion of distribution mainly occurs in the northwestern region of Guizhou, the central part of Sichuan and the junction zone of Sichuan, Shaanxi and Chongqing. The most significant expansion is observed during the ssp245 scenario in the 2050s period (Figure 6).

**FIGURE 8:**
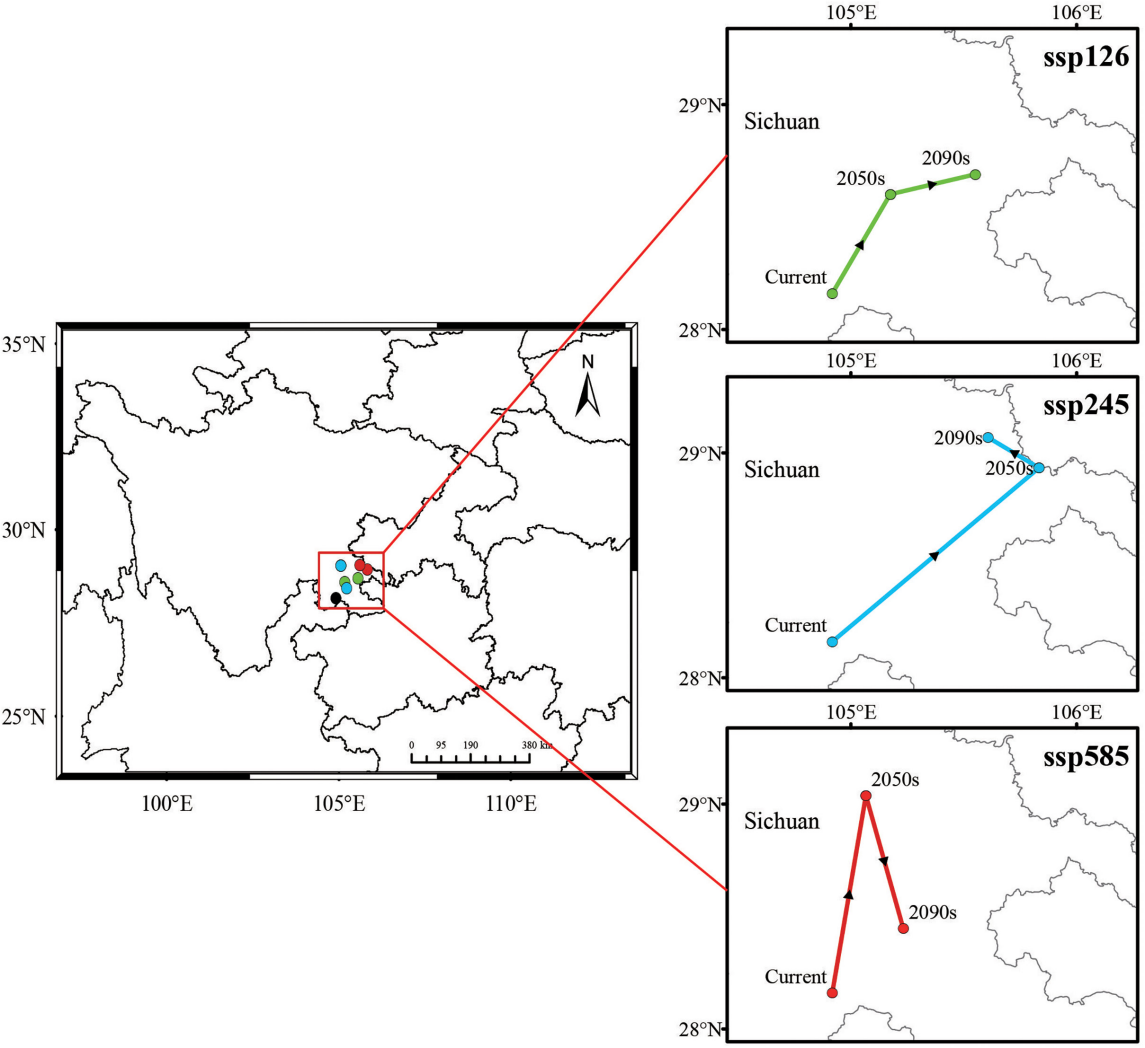
Shift of distribution centroid of *Chimonobambusa tumidissima* from current to future climate scenarios.

The current distribution center is situated at the border of Weixin in Yunnan province and Xuyong in Sichuan province (104°55′03.88″ E, 28°9′39.88″ N). With the progression of time and the intensification of climate conditions (from ssp126 to ssp585), the centroid of the suitable area is observed to be shifting towards the northeast compared to its current location (Figure 8). According to projections based on the ssp126 scenario, it is anticipated that the distribution center will migrate approximately 54.98 and 85.48 km by the 2050s and 2090s, respectively. A more substantial displacement is observed under the ssp245 scenario, with an estimated shift of around 123.94 km and 121.26 km compared to its current position. Under the ssp585 scenario, it is expected that there will be a northward migration of about 98.48 km by the 2050s and a shift of approximately 44.28 km towards northeast by the 2090s.

## 4 DISCUSSION

### 4.1 Environmental variables determining the distribution of *C. Tumidissinoda*

*C. tumidissinoda*, a dominant species found in the subalpine zone transitioning from Yunnan‐Guizhou Plateau to Sichuan Basin, typically exhibits a preference for mild and humid environmental conditions. The jackknife method elucidates that the distribution of *C. tumidissinoda* is minimally influenced by soil and slope, but predominantly affected by Bio14 (31.24%), elevation (28.27%), and Bio3 (17.24%), which are concordant with its specific habitat requirements. Li et al. (2019) reported that precipitation plays a pivotal role in determining the distribution of bamboo forests, followed closely by temperature. Additionally, Zhao et al. (2023) revealed that precipitation and temperature are crucial factors in the germination and development of bamboo shoots of *Bashania fargesii*, which serves as a primary food source for giant panda. It is worth noting that when the precipitation of the driest month (Bio14) falls below 7 mm, *C. tumidissinoda* is unable to survive. We have observed that both the germination process and rapid growth of *C. tumidissinoda* bamboo shoots coincide with the onset of the rainy season, indicating a highly sensitivity to precipitation conditions in its growth processes, similar to other bamboo species (Hu & He, 2020; Li et al., 2019; Zhao et al., 2023). Simultaneously, the growth of *C. tumidissinoda* is also susceptible to temperature and requires a narrow range for optimal development, characterized by an isothermality (Bio3) ranging from 27.69% to 32.35%. This indicates that significant fluctuations in temperature could have detrimental effects on its growth, consistent with the findings from study on tropical bamboo *Dendrocalamus sinicus* (Dou et al., 2022). Dong et al. (2002) have observed that the daily increase of *C. tumidissinoda* bamboo height is positively correlated with the daily mean temperature, further verifying that temperature is also a main factor affecting the rapid growth of this bamboo species.

Previous researches have demonstrated that elevation plays a crucial role in shaping the distribution patterns of montane plants (Du et al., 2024; Hu & He, 2020; Huan et al., 2023; Qiu, Fu, et al., 2023). Our simulation results further validate this relationship by confirming that elevation (28.27%) is one of the critical factors determining the distribution pattern of this particular bamboo species, similar to the bamboo species found in the Hengduan mountains (Hu & He, 2020). And the optimal altitude range for plant growth of *C. tumidissinoda* is observed between 1341 and 1905 m, which corresponds with its subalpine adaptive habitat.

### 4.2 Distribution range shift for *C. Tumidissinoda*

By employing the MaxEnt model, we successfully delineated the suitable habitat for *C. tumidissinoda* in Zhaotong, Yunnan province and its surrounding areas under prevailing climatic conditions. Previous studies have documented a tendency of montane plants to migrate towards higher latitudes in response to climate change (He, Burgess, Gao, & Li, 2019; Liang et al., 2018). The findings of this investigation revealed a similar phenomenon. The results indicate that within our research zone, the current distribution range of *C. tumidissinoda* spans from 27° N to 29° N, with its centroid located at latitude 28°9′39.88″ N. Under future climate scenarios, it is projected that there will be a shift towards northeast in the range of *C. tumidissinoda*. The estimated migration distance ranges from 44.28 to 123.94 km, with latitudes spanning from 28°26′50.44″ N to 29°4′6.06″ N. Historical demographical analyses have suggested that a cold and arid climate may contribute to the population decline of *C. tumidissinoda* (Ye et al., 2024). Regions with higher latitudes can potentially drive the geographical shift of this bamboo species by providing more humid and warmer climate conditions (Chen et al., 2011). Similarly, projections for alpine bamboo *Bashania fargesii* (Zhao et al., 2023) in the Qinling Mountains and African bamboo *Oxytenanthera abyssinica* (Gebrewahid et al., 2020) in Ethiopia also presented a shift towards higher latitudes. And this tendency has also been observed in other plant species such as *Dactylorhiza hatagirea* (Singh et al., 2022), *Meconopsis* (He, Burgess, Yang, et al., 2019), *Habenaria* (Qiu, Jacquemyn, et al., 2023) and *Senegalia senegal* (Fang et al., 2024).

Moreover, the adaptability of this bamboo species to future environments is expected to be higher under both moderate and pessimistic climate scenarios, with a significant potential for expansion ranging from 14.16% to 84.07%. Specifically, *C. tumidissinoda* is projected to colonize new habitats in the central region of Sichuan province and at the junction of Sichuan, Shaanxi and Chongqing under future climate scenarios, particularly during the ssp245 period of the 2050s (Figure 6). The increase in global temperature of this scenario may lead to a more humid climate, which could account for the expansion of *C. tumidissinoda* into these new habitats. Furthermore, bamboo species typically propagate through rhizomes and exhibit a robust capacity for asexual reproduction. Once established in a particular area, it possesses the potential to become a dominant species. This phenomenon could be attributed as an important factor contributing to its future expansion under changing climatic conditions. However, the suitable area of this bamboo will decrease by 7.96% under an optimistic climate change scenario of the 2090s, contrasting with its current size. This implies that extreme climate change does not necessarily lead to a reduction in the range of suitable areas and different species have varying ability to adapt to climate change. For example, Fang et al. (2024) reported that the suitable zone of *Senegalia senegal* will increase significantly under the pessimistic climate scenario when compared to other more optimistic scenarios. Wang et al. (2024) found that the potential suitable area of relict plant *Davidia involucrate* decreased under the ssp126 and ssp585 scenarios but increased under the ssp370 climate scenario (2070s). Therefore, the resilience to extreme climate change for different species needs to be analyzed case‐by‐case.

### 4.3 Suggestions for conservation and introduction strategies

Although there is an expansion of suitable area for *C. tumidissinoda* under future moderate and pessimistic climate scenarios, the total suitable habitats remain limited (ranging from 1.29 to 2.08 × 10^4^ km^2^), and are primarily concentrated in Zhaotong and adjacent areas. Population genomics analysis revealed that *C. tumidissinoda* exhibits a low level of genetic diversity (*H*_*O*_ = 0.085, *H*_*E*_ = 0.115, *π* = 0.116) and may have limited adaptability to future climate change (Ye et al., 2024). Furthermore, apart from the environmental factors considered in this study such as bioclimate, topography and soil properties, numerous interactive factors including human activities and physiological properties could potentially influence the growth of *C. tumidissinoda* as well. Given the increasing attention towards healthy eating habits and the growing popularity of green foods like bamboo shoots among people today, it is important to consider how human activities might impact the distribution of *C. tumidissinoda*. Additionally, the blooming of *C. tumidissinoda* can lead to plant mortality (Janzen, 1976), posing a further threat to its survival. Therefore, it is imperative to develop conservation strategies, and the regions currently exhibiting suitability for *C. tumidissinoda* and overlapping with the existing area can serve as in situ conservation sites. In these future scenarios, the central region of Sichuan province and the junction area of Sichuan, Shaanxi and Chongqing have emerged as newly identified highly suitable areas. However, due to limited dispersal capacity and fragmentation of these regions, natural colonization by *C. tumidissinoda* is unlikely and transplantation of individuals into these areas would be optimal. Based on the MaxEnt results, the southeast of Sichuan and northwest of Guizhou are predicted to be at high risk of disappearing in the future. Therefore, it is crucial to pay more attention to these areas and germplasm resources should be collected to avoid the loss of rare genes in these populations.

In addition, *C. tumidissinoda* has a high economic value for its elegant form and can be cultivated as ornamental plants. But unregulated introduction may lead to unsuccessful cultivation attempts. The distribution map presented in this study, which illustrates the suitable areas and crucial environmental factors for both current and future scenarios of *C. tumidissinoda*, can facilitate its controlled introduction and assist in urban cultivation planning. Specifically, the newly identified suitable habitats of *C. tumidissinoda* can be prioritized for introduction and cultivation.

## 5 CONCLUSION

In this study, we employed the MaxEnt model to predict the suitable areas of *C. tumidissinoda* under current and varying future climate scenarios. The precipitation of the driest month (Bio14), elevation and isothermality (Bio3) were identified as crucial environmental factors influencing the species' distribution. Under current conditions, only 0.6% of the research area exhibited suitability for its occurrence. Furthermore, significant disparities were observed in the species' responses to different climate changes. The projected habitat suitability for *C. tumidissinoda* is anticipated to contract or remain unchanged in the optimistic climate change scenario but expand in moderate and pessimistic climate scenarios. In response to future climate change, the potential range of *C. tumidissinoda* is projected to shift towards higher latitude and fragmented regions, such as central Sichuan and the junction region of Sichuan, Shaanxi and Chongqing. Furthermore, there will be a northeastward shift in the distribution centroid. Based on these distribution dynamics, regions that maintain their suitability from current to future climate scenarios may be serve as valuable climate refugia for *C. tumidissinoda* and should be prioritized for in situ conservation efforts. Meanwhile, the newly identified habitats can be utilized as ex situ conservation sites for introduction and cultivation. In conclusion, our findings establish a foundation for evaluating the adaptability of *C. tumidissinoda* to climate change, as well as for formulating effective strategies for conservation and sustainable utilization.

## AUTHOR CONTRIBUTIONS

**Wei‐Hua Wang:** Formal analysis (lead); methodology (equal); writing – original draft (lead). **Shu‐Lei Peng:** Methodology (equal); software (equal). **Hua Shu:** Investigation (lead); software (equal). **Xi Fu:** Data curation (lead). **Xia‐Ying Ye:** Funding acquisition (lead); supervision (lead); writing – original draft (supporting); writing – review and editing (lead).

## CONFLICT OF INTEREST STATEMENT

The authors declare no conflicts of interest.

## Supporting information


**Table S1:** Occurrence records of *Chimonobambusa tumidissinoda* utilized in this study.

## Data Availability

All necessary data have already been provided in the main text and supporting information.

## References

[ece370314-bib-0001] Blackburn, L. M. , Elkinton, J. S. , Havill, N. P. , Broadley, H. J. , Andersen, J. C. , & Liebhold, A. M. (2020). Predicting the invasion range for a highly polyphagous and widespread forest herbivore. NeoBiota, 59, 1–20. 10.3897/neobiota.59.53550

[ece370314-bib-0002] Brown, J. L. (2014). SDMtoolbox: A python‐based GIS toolkit for landscape genetic, biogeographic and species distribution model analyses. Methods in Ecology and Evolution, 5(7), 694–700. 10.1111/2041-210X.12200 PMC5721907 29230356

[ece370314-bib-0003] Chen, I. C. , Hill, J. K. , Ohlemüller, R. , Roy, D. B. , & Thomas, C. D. (2011). Rapid range shifts of species associated with high levels of climate warming. Science, 333(6045), 1024–1026. 10.1126/science.1206432 21852500

[ece370314-bib-0004] Dai, E. C. , Zhao, X. Y. , He, X. H. , & Wang, S. (2023). Cloning and expression analysis of the *QtKQB1* gene under abiotic stress in *Qiongzhuea tumidinoda* . Molecular Plant Breeding. https://link.cnki.net/urlid/46.1068.S.20231218.1428.006

[ece370314-bib-0005] Dong, W. Y. (2006). Studies on the clonal population degradation and restoration mechanism of Qiongzhuea tumidinoda. Postdoctor work report. The Chinese Academy of Forestry.

[ece370314-bib-0006] Dong, W. Y. , Huang, B. L. , Xie, Z. X. , Xie, Z. H. , & Liu, H. Y. (2002). The study on the growth and development rhythm of *Qiongzhuea tumidinoda* . Journal of Nanjing Forestry University (Natural Sciences Edition), 26(3), 43–46. 10.3969/j.jssn.1000-2006.2002.03.012

[ece370314-bib-0007] Dong, W. Y. , Qiu, Y. Q. , Wang, Y. Z. , & Yang, Y. (2016). Morphological genetic diversity of natural *Qiongzhuea tumidinoda* populations. Journal of Northeast Forestry University, 44(5), 101–103. 10.13759/j.cnki.dlxb.20160509.010

[ece370314-bib-0008] Dou, P. , Dong, Y. , Chen, L. , & Yang, H. Q. (2022). Modeling the potential distribution of different types of *Dendrocalamus sinicus*, the strongest woody bamboo in the world, with MaxEnt model. PeerJ, 10, e13847. 10.7717/peerj.13847 35935247 PMC9354798

[ece370314-bib-0009] Du, B. Y. , Wang, Z. Q. , Li, X. Y. , Zhang, X. , Wang, X. T. , & Zhang, D. Y. (2024). Adaptation of tree species in the greater Khingan range under climate change: Ecological strategy differences between *Larix gmelinii* and *Quercus mongolica* . Forests, 15, 283. 10.3390/f15020283

[ece370314-bib-0010] Elith, J. , Graham, C. H. , Anderson, R. P. , Dudík, M. , Ferrier, S. , Guisan, A. , Hijmans, R. J. , Huettmann, F. , Leathwick, J. R. , & Lehmann, A. (2006). Novel methods improve prediction of species' distributions from occurrence data. Ecography, 29(2), 129–151. 10.1111/j.2006.0906-7590.04596.x

[ece370314-bib-0011] Fang, J. Q. , Shi, J. F. , Zhang, P. , Shao, M. H. , Zhou, N. , Wang, Y. D. , & Xu, X. W. (2024). Potential distribution projections for *Senegalia Senegal* (L.) Britton under climate change scenarios. Forests, 15(2), 379. 10.3390/f15020379

[ece370314-bib-0012] Fick, S. E. , & Hijmans, R. J. (2017). WorldClim 2: New 1‐km spatial resolution climate surfaces for global land areas. International Journal of Climatology, 37(12), 4302–4315. 10.1002/joc.5086

[ece370314-bib-0013] Fu, L. G. (1991). China plant red data book: Rare and endangered plants. Science Press.

[ece370314-bib-0014] Gao, P. H. , Li, Y. , & Yan, B. (2021). Cloning and expression analysis of *QtKAT1* gene in *Qiongzhuea tumidinoda* . Molecular Plant Breeding, 19(4), 1113–1120. 10.13271/j.mpb.019.001113

[ece370314-bib-0015] Gao, X. , Liu, J. , & Huang, Z. (2022). The impact of climate change on the distribution of rare and endangered tree *Firmiana kwangsiensis* using the Maxent modeling. Ecology and Evolution, 12(8), e9165. 10.1002/ece3.9165 35919389 PMC9336174

[ece370314-bib-0016] Gebrewahid, Y. , Abrehe, S. , Meresa, E. , Eyasu, G. , Abay, K. , Gebreab, G. , Kidanemariam, K. , Adissu, G. , Abreha, G. , & Darcha, G. (2020). Current and future predicting potential areas of *Oxytenanthera abyssinica* (a. Richard) using MaxEnt model under climate change in northern Ethiopia. Ecological Processes, 9(1), 6. 10.1186/s13717-019-0210-8

[ece370314-bib-0017] Guisan, A. , & Zimmermann, N. E. (2000). Predictive habitat distribution models in ecology. Ecological Modelling, 135, 147–186.

[ece370314-bib-0018] He, X. , Burgess, K. S. , Gao, L. M. , & Li, D. Z. (2019). Distributional responses to climate change for alpine species of *Cyananthus* and *primula* endemic to the Himalaya‐Hengduan Mountains. Plant Diversity, 41(1), 26–32. 10.1016/j.pld.2019.01.004 30931415 PMC6412159

[ece370314-bib-0019] He, X. , Burgess, K. S. , Yang, X. F. , Ahrends, A. , Gao, L. M. , & Li, D. Z. (2019). Upward elevation and northwest range shifts for alpine *Meconopsis* species in the Himalaya‐Hengduan Mountains region. Ecology and Evolution, 9(7), 4055–4064. 10.1002/ece3.5034 31015987 PMC6467849

[ece370314-bib-0020] Hu, S. P. , & He, L. W. (2020). Analysis of suitable distribution areas of *Fargesia denudata* in Baishuijiang National Nature Reserve using MaxEnt model and ArcGIS. Chinese Journal of Ecology, 6, 2115–2122. 10.13292/j.1000-4890.202006.023

[ece370314-bib-0021] Huan, Z. Q. , Geng, X. M. , Xu, X. R. , Liu, W. , Zhu, Z. L. , & Tang, M. (2023). Potential geographical distribution of *Michelia martinii* under different climate change scenarios based on MaxEnt model. Journal of Ecology and Rural Environment, 39(10), 1277–1287. 10.19741/j.issn.1673-4831.2022.0145

[ece370314-bib-0022] Hughes, A. C. (2017). Mapping priorities for conservation in Southeast Asia. Biological Conservation, 209, 395–405. 10.1016/j.biocon.2017.03.007

[ece370314-bib-0023] IPCC . (2023). Climate change 2023: Synthesis report. Contribution of working groups I, II and III to the sixth assessment report of the intergovernmental panel on climate change. IPCC.

[ece370314-bib-0024] Janzen, D. H. (1976). Why bamboos wait so long to flower. Annual Review of Ecology and Systematics, 7, 347–391.

[ece370314-bib-0025] Jia, W. J. , & Wang, S. (2021). Germination conditions of *Qiongzhuea tumidinoda* seed and mineral element analysis of *Qiongzhuea tumidinoda* shoots. Seed, 40(1), 79–83. 10.16590/j.cnki.1001-4705.2021.01.079

[ece370314-bib-0026] Keng, P. C. , & Wang, Z. P. (1996). Flora Reipublicae Popularis Sinicae. Science Press.

[ece370314-bib-0027] Li, D. Z. , Wang, Z. P. , Zhu, Z. D. , Xia, N. H. , Jia, L. Z. , Guo, Z. H. , Yang, G. Y. , & Stapleton, C. M. A. (2006). Tribe Bambuseae. In Z. Y. Wu , P. H. Raven , & D. Y. Hong (Eds.), Flora of China (Vol. 22, pp. 7–180). Science Press, Beijing and Missouri Botanical Garden Press.

[ece370314-bib-0028] Li, L. F. , Liu, W. N. , Ai, J. W. , Cai, S. J. , & Dong, J. W. (2023). Predicting mangrove distributions in the Beibu gulf, Guangxi, China, using the MaxEnt model: Determining tree species selection. Forests, 14(1), 149. 10.3390/f14010149

[ece370314-bib-0029] Li, X. J. , Mao, F. J. , Du, H. Q. , Zhou, G. M. , Xing, L. Q. , Liu, T. Y. , Han, N. , Liu, Y. L. , Zhu, D. E. , Zheng, J. L. , Dong, L. F. , & Zhang, M. (2019). Spatiotemporal evolution and impacts of climate change on bamboo distribution in China. Journal of Environmental Management, 248, 109265. 10.1016/j.jenvman.2019.109265 31352276

[ece370314-bib-0030] Li, Y. , Xiao, R. X. , Rui, R. , & Wang, S. (2021). Cloning and expression analysis on *QtNHX1* gene from *Qiongzhuea tumidinoda* . Molecular Plant Breeding, 19(10), 3235–3242. 10.13271/j.mpb.019.003235

[ece370314-bib-0031] Liang, Q. L. , Xu, X. T. , Mao, K. S. , Wang, M. C. , Wang, K. , Xi, Z. X. , & Liu, J. Q. (2018). Shifts in plant distributions in response to climate warming in a biodiversity hotspot, the Hengduan Mountains. Journal of Biogeography, 45(6), 1334–1344. 10.1111/jbi.13229

[ece370314-bib-0032] Mastrandrea, M. D. , Field, C. B. , Stocker, T. F. , Edenhofer, O. , Ebi, K. L. , Frame, D. J. , Held, H. , Kriegler, E. , Mach, K. J. , Matschoss, P. R. , Plattner, G.‐K. , Yohe, G. W. , & Zwiers, F. W. (2010). Guidance note for lead authors of the IPCC fifth assessment report on consistent treatment of uncertainties. Intergovernmental Panel on Climate Change (IPCC). https://www.ipcc.ch

[ece370314-bib-0033] McCulloch‐Jones, E. J. , Kraaij, T. , Crouch, N. , & Faulkner, K. T. (2023). Assessing the invasion risk of traded alien ferns using species distribution models. NeoBiota, 87, 161–189. 10.3897/neobiota.87.101104

[ece370314-bib-0034] Ohlemuller, R. , Anderson, B. J. , Araujo, M. B. , Butchart, S. H. , Kudrna, O. , Ridgely, R. S. , & Thomas, C. D. (2008). The coincidence of climatic and species rarity: High risk to small‐range species from climate change. Biology Letters, 4(5), 568–572. 10.1098/rsbl.2008.0097 18664421 PMC2610064

[ece370314-bib-0035] Phillips, S. J. , Anderson, R. P. , & Schapire, R. E. (2006). Maximum entropy modeling of species geographic distributions. Ecological Modelling, 190(3–4), 231–259. 10.1016/j.ecolmodel.2005.03.026

[ece370314-bib-0036] Qiu, L. , Fu, Q. L. , Jacquemyn, H. , Burgess, K. S. , Cheng, J. J. , Mo, Z. Q. , Tang, X. D. , Yang, B. Y. , & Tan, S. L. (2023). Contrasting range changes of *Bergenia* (Saxifragaceae) species under future climate change in the Himalaya and Hengduan Mountains region. Theoretical and Applied Climatology, 155(3), 1927–1939. 10.1007/s00704-023-04746-0

[ece370314-bib-0037] Qiu, L. , Jacquemyn, H. , Burgess, K. S. , Zhang, L. G. , Zhou, Y. D. , Yang, B. Y. , & Tan, S. L. (2023). Contrasting range changes of terrestrial orchids under future climate change in China. Science of the Total Environment, 895, 165128. 10.1016/j.scitotenv.2023.165128 37364836

[ece370314-bib-0038] Qiu, Y. Q. , Dong, W. Y. , Wang, Y. Z. , & Fu, J. S. (2017). Genetic diversity in natural populations of *Qiongzhuea tumidinoda* . Journal of Northwest Foresty University, 32(2), 155–160. 10.3969/j.issn.1001-7461.2017.02.26

[ece370314-bib-0039] Radosavljevic, A. , & Anderson, R. P. (2013). Making better Maxent models of species distributions: Complexity, overfitting and evaluation. Journal of Biogeography, 41(4), 629–643. 10.1111/jbi.12227

[ece370314-bib-0040] Rawien, J. , & Jairam‐Doerga, S. (2022). Predicted *Batrachochytrium dendrobatidis* infection sites in Guyana, Suriname, and French Guiana using the species distribution model MaxEnt. PLoS One, 17(7), e0270134. 10.1371/journal.pone.0270134 35834475 PMC9282542

[ece370314-bib-0041] Rong, S. T. , Luo, P. R. , Yi, H. , Yang, X. , Zhang, L. H. , Zeng, D. , & Wang, L. (2023). Predicting habitat suitability and adaptation strategies of an endangered endemic species, *camellia luteoflora* Li ex Chang (Ericales: Theaceae) under future climate change. Forests, 14(11), 2177. 10.3390/f14112177

[ece370314-bib-0042] Ru, G. X. , Yuan, J. L. , Zhang, D. , & Guo, G. P. (2010). Analysis of populations genetic diversity of *Qiongzhuea tumidinoda* using AFLP markers. Forest Research, 23(6), 850–855.

[ece370314-bib-0043] Shi, X. D. , Yin, Q. , Sang, Z. Y. , Zhu, Z. L. , Jia, Z. K. , & Ma, L. Y. (2023). Habitat distribution pattern of rare and endangered plant *Magnolia wufengensis* in China under climate change. Forests, 14(9), 1767. 10.3390/f14091767

[ece370314-bib-0044] Shi, Y. H. , Ren, Z. X. , Wang, W. J. , Xu, X. , Liu, J. , Zhao, Y. H. , & Wang, H. (2021). Predicting the spatial distribution of three *Astragalus* species and their pollinating bumblebees in the Sino‐Himalayas. Biodiversity Science, 29(6), 759–769. 10.17520/biods.2020268

[ece370314-bib-0045] Singh, L. , Kanwar, N. , Bhatt, I. D. , Nandi, S. K. , & Bisht, A. K. (2022). Predicting the potential distribution of *Dactylorhiza hatagirea* (D. Don) Soo‐an important medicinal orchid in the west Himalaya, under multiple climate change scenarios. PLoS One, 17(6), e0269673. 10.1371/journal.pone.0269673 35714160 PMC9205508

[ece370314-bib-0046] Swets, J. A. (1988). Measuring the accuracy of diagnostic systems. Science, 240(4857), 1285–1293. 10.1126/science.3287615 3287615

[ece370314-bib-0047] Tang, S. L. , Song, Y. B. , Zeng, B. , & Dong, M. (2022). Potential distribution of the extremely endangered species *Ostrya rehderiana* (Betulaceae) in China under future climate change. Environmental Science and Pollution Research, 29(5), 7782–7792. 10.1007/s11356-021-16268-1 34476707

[ece370314-bib-0048] Thapa, A. , Wu, R. , Hu, Y. , Nie, Y. , Singh, P. B. , Khatiwada, J. R. , Yan, L. , Gu, X. , & Wei, F. (2018). Predicting the potential distribution of the endangered red panda across its entire range using MaxEnt modeling. Ecology and Evolution, 8(21), 10542–10554. 10.1002/ece3.4526 30464826 PMC6238126

[ece370314-bib-0049] Tu, W. Q. , Xiong, Q. L. , Qiu, X. P. , & Zhang, Y. M. (2021). Dynamics of invasive alien plant species in China under climate change scenarios. Ecological Indicators, 129, 107919. 10.1016/j.ecolind.2021.107919

[ece370314-bib-0050] Vasconcelos, T. S. , Rodríguez, M. Á. , & Hawkins, B. A. (2011). Species distribution modelling as a macroecological tool: A case study using New World amphibians. Ecography, 35(6), 539–548. 10.1111/j.1600-0587.2011.07050.x

[ece370314-bib-0051] Wang, L. , Dong, W. Y. , Zhao, J. F. , & Mao, W. J. (2012). Biodiversity of *Qiongzhuea tumidinosa* community in Daguan county of Yunnan province. Journal of West China Forestry Science, 41(3), 60–65. 10.16473/j.cnki.xblykx1972.2012.03.021

[ece370314-bib-0052] Wang, T. , Li, W. , Cui, H. , Song, Y. , Liu, C. , Yan, Q. , Wu, Y. , Jia, Y. , Fang, L. , & Qi, L. (2024). Predicting the potential habitat distribution of relict plant *Davidia involucrata* in China based on the MaxEnt model. Forests, 15(2), 272. 10.3390/f15020272

[ece370314-bib-0053] Wang, W. H. , Yang, X. Q. , Yang, S. Q. , Zhao, P. , Liu, Y. , Zhu, G. L. , & Ye, X. Y. (2023). Comparative metabolomics revealed metabolite difference of bamboo shoots (*Chimonobambusa tumidissinoda* Hsueh & T. P. Yi ex Ohrnberger) sampled from different growth stages. Food Science, 44(14), 237–244. 10.7506/spkx1002-6630-20220926-278

[ece370314-bib-0054] Wu, Y. Y. , Dong, W. Y. , Liu, P. , Zhang, M. N. , Xie, Z. X. , & Tian, F. K. (2020). Anatomical characteristics and adaptability plasticity of *Qiongzhuea tumidinoda* stalk under different soil water and nutrient conditions. Journal of Beijing Forestry University, 42(4), 80–90. 10.12171/j.1000-1522.20190290

[ece370314-bib-0055] Wu, Y. Y. , Dong, W. Y. , Yi, Z. N. , Zhen, J. N. , Ren, Z. J. , Xie, Z. X. , & Tian, F. K. (2019). Effects of different slope positions on *Qiongzhuea tumidinoda* clone population growth. World Bamboo and Rattan, 17(2), 22–25. 10.13640/j.cnki.wbr.2019.02.005

[ece370314-bib-0056] Yan, M. X. , Gu, B. J. , Zhang, M. X. , Wang, W. , Quan, R. C. , Li, J. Q. , & Wang, L. (2021). The range contraction and future conservation of green peafowl (*Pavo muticus*) in China. Sustainability, 13(21), 11723. 10.3390/su132111723

[ece370314-bib-0057] Yan, X. , Wang, S. , Duan, Y. , Han, J. , Huang, D. , & Zhou, J. (2021). Current and future distribution of the deciduous shrub *Hydrangea macrophylla* in China estimated by MaxEnt. Ecology and Evolution, 11(22), 16099–16112. 10.1002/ece3.8288 34824814 PMC8601876

[ece370314-bib-0058] Yang, Y. , Dong, W. Y. , Que, Y. Q. , Li, W. , Yang, J. J. , & Han, Y. (2015). Transformation of nutritional composition in *Chimonobambusa tumidissinoda* shoots during growth process. Journal of Northeast Forestry University, 43(1), 80–87. 10.13759/j.cnki.dlxb.2015.01.004

[ece370314-bib-0059] Ye, X. Y. , Wang, W. H. , Wei, G. R. , Li, B. , Li, Y. , & Ma, P. F. (2024). Genetic structure of a narrowly distributed species *Chimonobambusa tumidissinoda* in the Yunnan‐Guizhou plateau, and its implications for conservation. Global Ecology and Conservation, 53, e03028. 10.1016/j.gecco.2024.e03028

[ece370314-bib-0060] Yuan, J. L. , Xiong, D. G. , Hu, B. T. , Jin, G. , Zhong, Z. Q. , Huang, L. J. , & Ma, N. X. (2008). Study on shoot nutrition of *Qiongzhuea tumidinoda*: A rare and protected bamboo species. Forest Research, 21(6), 773–777.

[ece370314-bib-0061] Zhang, H. Y. , Sun, P. F. , Zou, H. C. , Ji, X. D. , Wang, Z. Y. , & Liu, Z. (2024). Adaptive distribution and vulnerability assessment of endangered maple species on the Tibetan plateau. Forests, 15(3), 491. 10.3390/f15030491

[ece370314-bib-0062] Zhang, Q. , Shen, X. B. , Jiang, X. L. , Fan, T. T. , Liang, X. C. , & Yan, W. D. (2023). MaxEnt modeling for predicting suitable habitat for endangered tree *Keteleeria davidiana* (Pinaceae) in China. Forests, 14(2), 394. 10.3390/f14020394

[ece370314-bib-0063] Zhang, W. , Dong, W. Y. , Zhong, H. , Li, J. , Liu, Z. L. , Wu, Y. Y. , & Pu, C. (2020). Effects of different mixed types on the growth of *Qiongzhuea tumidinoda* and spatial differences of soil nutrients. Journal of West China Forestry Science, 49(6), 70–75. 10.16473/j.cnki.xblykx1972.2020.06.010

[ece370314-bib-0064] Zhao, H. R. , Yang, X. T. , Shi, S. Y. , Xu, Y. D. , Yu, X. P. , & Ye, X. P. (2023). Climate‐driven distribution changes for *Bashania fargesii* in the Qinling Mountains and its implication for panda conservation. Global Ecology and Conservation, 46, e02610. 10.1016/j.gecco.2023.e02610

[ece370314-bib-0065] Zheng, J. N. , Dong, W. Y. , Zhong, H. , Yi, Z. N. , & Wu, Y. Y. (2021). Technical research on rapid propagation of *Qiongzhuea tumidinoda* tissue culture. Journal of Northeast Forestry University, 49(1), 50–59. 10.13759/j.cnki.dlxb.2021.01.010

[ece370314-bib-0066] Zhong, H. , Dong, W. Y. , Pu, C. , Xie, Z. X. , Zhang, W. , Zheng, J. N. , & Xia, L. (2023). Ecological stoichiometry of soil C, N and P in 4 different types of *Qiongzhuea tumidinoda* forests in Northeast Yunnan. Journal of Southwest Forestry University (Natural Science), 43(3), 111–119. 10.11929/j.swfu.202203054

[ece370314-bib-0067] Zhou, R. B. , Gao, Y. , Chang, N. , Gao, T. , Ma, D. L. , Li, C. , & Liu, Q. Y. (2021). Projecting the potential distribution of *Glossina morsitans* (Diptera: Glossinidae) under climate change using the MaxEnt model. Biology (Basel), 10(11), 1150. 10.3390/biology10111150 34827144 PMC8615152

