# Supplementary material for: Potential adaptive habitats for the narrowly distributed and rare bamboo species Chimonobambusa tumidissinoda J. R. Xue & T. P. Yi ex Ohrnb. under future climate change in China
Source: Ecol Evol. 2024 Sep 15;14(9):e70314. doi: 10.1002/ece3.70314 (PMC11402478; doi:10.1002/ece3.70314)
Supplement: Supplementary file 1 — Table S1: Occurrence records of Chimonobambusa tumidissinoda utilized in this study. [file ECE3-14-e70314-s001.docx]

Table S1 Occurrence records of *Chimonobambusa tumidissinoda* utilized in this study

| Species | Latitude | Longitude |
| --- | --- | --- |
| *Chimonobambusa tumidissinoda* | 27.883 | 104.677 |
| *Chimonobambusa tumidissinoda* | 27.878 | 104.672 |
| *Chimonobambusa tumidissinoda* | 27.882 | 104.708 |
| *Chimonobambusa tumidissinoda* | 27.897 | 104.740 |
| *Chimonobambusa tumidissinoda* | 28.245 | 103.982 |
| *Chimonobambusa tumidissinoda* | 28.256 | 103.982 |
| *Chimonobambusa tumidissinoda* | 28.238 | 103.986 |
| *Chimonobambusa tumidissinoda* | 28.220 | 103.931 |
| *Chimonobambusa tumidissinoda* | 28.227 | 103.930 |
| *Chimonobambusa tumidissinoda* | 28.236 | 103.927 |
| *Chimonobambusa tumidissinoda* | 28.241 | 103.920 |
| *Chimonobambusa tumidissinoda* | 28.211 | 103.938 |
| *Chimonobambusa tumidissinoda* | 28.212 | 104.001 |
| *Chimonobambusa tumidissinoda* | 28.220 | 104.005 |
| *Chimonobambusa tumidissinoda* | 27.227 | 103.645 |
| *Chimonobambusa tumidissinoda* | 27.847 | 104.305 |
| *Chimonobambusa tumidissinoda* | 28.341 | 103.734 |
| *Chimonobambusa tumidissinoda* | 28.346 | 103.744 |
| *Chimonobambusa tumidissinoda* | 28.132 | 103.976 |
| *Chimonobambusa tumidissinoda* | 28.114 | 104.000 |
| *Chimonobambusa tumidissinoda* | 28.142 | 103.979 |
| *Chimonobambusa tumidissinoda* | 27.999 | 105.160 |
| *Chimonobambusa tumidissinoda* | 28.006 | 105.173 |
| *Chimonobambusa tumidissinoda* | 27.546 | 105.036 |
| *Chimonobambusa tumidissinoda* | 27.861 | 104.956 |
| *Chimonobambusa tumidissinoda* | 27.700 | 104.850 |
| *Chimonobambusa tumidissinoda* | 27.752 | 104.862 |
| *Chimonobambusa tumidissinoda* | 28.048 | 103.868 |
| *Chimonobambusa tumidissinoda* | 27.722 | 103.932 |
| *Chimonobambusa tumidissinoda* | 27.891 | 104.757 |
| *Chimonobambusa tumidissinoda* | 28.418 | 104.124 |
| *Chimonobambusa tumidissinoda* | 28.411 | 104.148 |
| *Chimonobambusa tumidissinoda* | 28.415 | 104.159 |
| *Chimonobambusa tumidissinoda* | 28.380 | 104.068 |
| *Chimonobambusa tumidissinoda* | 27.920 | 104.055 |
| *Chimonobambusa tumidissinoda* | 27.927 | 104.049 |
| *Chimonobambusa tumidissinoda* | 28.300 | 103.600 |
| *Chimonobambusa tumidissinoda* | 27.700 | 103.900 |
| *Chimonobambusa tumidissinoda* | 27.894 | 104.716 |
| *Chimonobambusa tumidissinoda* | 27.913 | 105.285 |
| *Chimonobambusa tumidissinoda* | 28.200 | 105.400 |
| *Chimonobambusa tumidissinoda* | 28.100 | 105.500 |
| *Chimonobambusa tumidissinoda* | 27.751 | 104.840 |
| *Chimonobambusa tumidissinoda* | 27.918 | 105.240 |
| *Chimonobambusa tumidissinoda* | 27.884 | 104.783 |
| *Chimonobambusa tumidissinoda* | 27.753 | 104.300 |
| *Chimonobambusa tumidissinoda* | 28.264 | 103.982 |
| *Chimonobambusa tumidissinoda* | 28.263 | 103.572 |
| *Chimonobambusa tumidissinoda* | 28.109 | 104.234 |
| *Chimonobambusa tumidissinoda* | 28.315 | 103.447 |
| *Chimonobambusa tumidissinoda* | 27.395 | 104.930 |
| *Chimonobambusa tumidissinoda* | 27.537 | 104.429 |
| *Chimonobambusa tumidissinoda* | 28.136 | 103.546 |
| *Chimonobambusa tumidissinoda* | 28.233 | 103.905 |
| *Chimonobambusa tumidissinoda* | 27.441 | 104.870 |
| *Chimonobambusa tumidissinoda* | 28.445 | 104.142 |
